# Recent Advances of Functional Proteomics in Gastrointestinal Cancers- a Path towards the Identification of Candidate Diagnostic, Prognostic, and Therapeutic Molecular Biomarkers

**DOI:** 10.3390/ijms21228532

**Published:** 2020-11-12

**Authors:** Morteza Abyadeh, Anna Meyfour, Vivek Gupta, Masoud Zabet Moghaddam, Matthew J. Fitzhenry, Shila Shahbazian, Ghasem Hosseini Salekdeh, Mehdi Mirzaei

**Affiliations:** 1 Cell Science Research Center, Department of Molecular Systems Biology, Royan Institute for Stem Cell Biology and Technology, ACECR, Tehran 1665659911, Iran; mabyadeh94@gmail.com (M.A.); hsalekdeh@yahoo.com (G.H.S.); 2 Basic and Molecular Epidemiology of Gastrointestinal Disorders Research Center, Research Institute for Gastroenterology and Liver Diseases, Shahid Beheshti University of Medical Sciences, Tehran 1985717413, Iran; 3 Cell Science Research Center, Department of Stem Cells and Developmental Biology, Royan Institute for Stem Cell Biology and Technology, ACECR, Tehran 1665659911, Iran; 4 Department of Clinical Medicine, Macquarie University, Macquarie Park, NSW 2113, Australia; vivek.gupta@mq.edu.au; 5 Center for Biotechnology and Genomics, Texas Tech University, Lubbock, TX 79409, USA; masoud.zabet@ttu.edu; 6 Australian Proteome Analysis Facility, Macquarie University, Macquarie Park, NSW 2113, Australia; matthew.fitzhenry@mq.edu.au; 7 Department of Molecular Sciences, Macquarie University, Macquarie Park, NSW 2113, Australia; shila.shahbazian@thermofisher.com

**Keywords:** phosphoproteomics, glycoproteomics, colorectal cancer, gastric cancer, hepatocellular carcinoma, pancreatic cancer

## Abstract

Gastrointestinal (GI) cancer remains one of the common causes of morbidity and mortality. A high number of cases are diagnosed at an advanced stage, leading to a poor survival rate. This is primarily attributed to the lack of reliable diagnostic biomarkers and limited treatment options. Therefore, more sensitive, specific biomarkers and curative treatments are desirable. Functional proteomics as a research area in the proteomic field aims to elucidate the biological function of unknown proteins and unravel the cellular mechanisms at the molecular level. Phosphoproteomic and glycoproteomic studies have emerged as two efficient functional proteomics approaches used to identify diagnostic biomarkers, therapeutic targets, the molecular basis of disease and mechanisms underlying drug resistance in GI cancers. In this review, we present an overview on how functional proteomics may contribute to the understanding of GI cancers, namely colorectal, gastric, hepatocellular carcinoma and pancreatic cancers. Moreover, we have summarized recent methodological developments in phosphoproteomics and glycoproteomics for GI cancer studies.

## 1. Introduction

Cancer is the third most leading cause of death worldwide after ischemic heart disease and stroke. Cancer related deaths were estimated to be approximately 9.6 million in 2018 [1], of those, 3.4 million deaths (more than one-third of all deaths) were caused by gastrointestinal (GI) cancers. Unfortunately the global GI cancer burden tends to increase to about 4.8 million new diagnosed cases in 2018 [2]. Three types of GI cancers including colorectal, stomach and liver are among the five most common causes of cancer related death globally [3]. The clinical management of GI cancers has remained a major challenge for clinicians.

GI cancers are almost always diagnosed at advanced stages which are associated with low survival rates. This is due to the lack of reliable biomarkers for early diagnosis or for screening high risk populations [4]. Moreover, disease mechanisms are not well understood which undermines therapeutic strategies. Therefore, finding reliable biomarkers and therapeutic targets are highly desirable and attract a great deal of attention among researchers. High-throughput omics technologies have provided the ability to investigate the molecular mechanisms of GI cancers at an unprecedented pace and in detail. Numerous genomic, transcriptomic, proteomic and metabolomic studies have been performed so far [5,6,7].

Proteome investigations are divided into two major areas: expression proteomics, which focuses on the down- and up-regulation of protein levels, and functional proteomics, which focuses on two major targets: discovering biological functions of unknown proteins and the definition of molecular mechanisms [8], including the characterization of enzymes activities, multiprotein complexes and post-translational modifications (PTMs) at proteins [9].

During the last decade, analyzing the alteration in post-translational modifications has received special interest as an efficient approach for biomarker discovery, putative therapeutic targets and understanding the disease mechanisms in GI cancers.

PTMs of proteins are one of the major cellular regulatory mechanisms affecting protein function, folding, interactions, localization, stability and turnover [9]. Amongst wide range protein PTMs, the most common forms of modifications include phosphorylation, glycosylation, methylation, acetylation, amidation, hydroxylation, sulfation, oxidation and ubiquitinylation.

Several proteomics studies focusing on PTMs in GI cancers have been published so far; including protein phosphorylation [10], glycosylation [11], acetylation [12], methylation [13] and ubiquitination [14]. Protein phosphorylation and glycosylation in particular have been extensively studied in GI cancers because of their key role in regulating a wide range of cellular signaling pathways associated with initiation, promotion and progression of the disease. Protein phosphorylation is one of the major types of post-translational modification (PTM) that mediates numerous physiological and pathological processes governed by 518 kinases and 226 protein phosphatases in humans [15]. Protein phosphorylation primarily occurs on serine, threonine and tyrosine residues and the aberrant phosphorylation of critical regulatory proteins drives numerous molecular mechanisms during tumorigenesis [16], suggesting kinases as the therapeutic targets and phosphorylation-related events as reliable biomarkers.

Glycosylation is another crucial form of protein post-translational modification that exists in about 50% of mammalian proteins [17]. Protein glycosylation plays a pivotal role in many biological pathways and molecular functions including cell–cell recognition, communication and adhesion, and has been shown to be altered during the process of tumor development and progression [4], therefore providing an interesting field for cancer biomarker discovery.

This review summarizes the recent phosphoproteomic and glycoproteomic studies in colorectal cancer, gastric cancer, hepatocellular carcinoma and pancreatic cancer. Furthermore, a comprehensive overview of developed strategies in these fields is also provided.

## 2. Quantitative Phosphoproteomics and Glycoproteomics Strategies

### 2.1. Phosphoproteomics

Phosphopeptide or phosphorylation site quantification is a valuable tool to understand the role of protein phosphorylation in cellular processes. Traditional approaches for exploring protein phosphorylation were laborious and time consuming such as radiolabeling of phosphorus atoms, phosphospecific antibodies and in-vitro kinase assays. These methods resulted in the identification of a limited number of phosphoproteins. The advent of high throughput mass spectrometry based methods has enabled the comprehensive study of phosphoproteins and led to the identification of a greater number of phosphoproteins.

Common methods for phosphoprotein analysis consist of three main steps: Phosphopeptide enrichment, peptide separation by HPLC followed by MS analysis.

**Figure 1:**
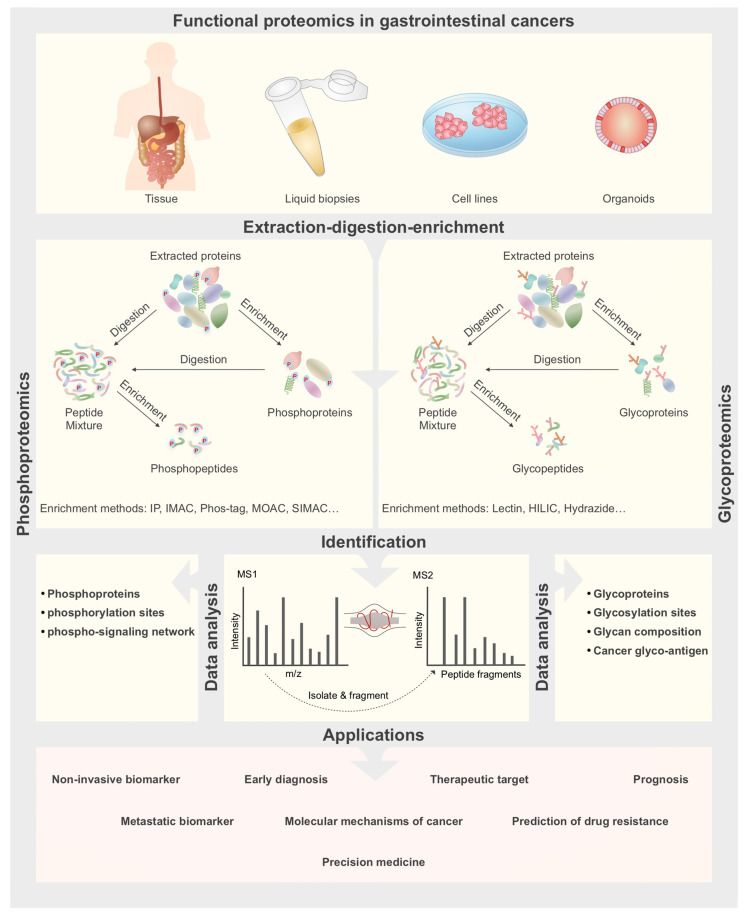
General workflow for functional proteomics analyses in gastrointestinal (GI) cancers. Samples can be obtained from patient’s tumor biopsies, body fluids, cultured cell models or patient-derived organoids. Samples are then subjected to protein extraction and enrichment for phosphoproteins and glycoproteins analyses, while for phosphopeptides and glycopeptides analyses an enzymatic digestion step is also carried out before or after enrichment. Then enriched samples are analyzed by LC-MS/MS to identify phosphoproteins, phosphopeptides, glycoproteins and glycopeptides. Finally results are used for biomarker discovery for cancer early diagnosis, prognosis and metastasis, and also to find therapeutic targets and drug resistance prediction.

Enrichment strategies can be applied on two levels, at the phosphoprotein level or at the phosphopeptide level [18,19] (Figure 1). However, phosphopeptide enrichment is more common [20]. The most common methods for enrichment of phosphoproteins or phosphopeptides include:

Phosphospecific antibodies (immunoprecipitation): in this method, antibodies are used against phosphorylated amino acids. Phosphoamino acid-selective antibodies are well suited to specifically identifying phosphoserine (pS) and phosphothreonine (pT) amino acids, although it is suitable for the whole phosphoproteome analysis. It is mainly applicable when one specific phosphorylated amino acid is being targeted and is not as useful for general phosphopeptide enrichment protocols [20,21,22,23].

Immobilized metal affinity chromatography (IMAC): this method primarily depends on the interaction between metal-ligand complexes and the specific functional groups in the protein. For both phosphopeptide and phosphoprotein enrichment, nitrilotriacetic acid (NTA) and iminodiacetic acid (IDA) are commonly used resins for IMAC. The metal ions that are commonly used are Fe^3+^, Ga^3+^, Zr^4+^ and Ti^4+^ for phosphopeptide binding. All these metal ions are positively charged, and are capable of binding negatively charged phosphate groups. Although IMAC has been used in a broad range of separations, it is less specific and selective compared to other affinity methods. Another challenge with this method is that the metal ions are not covalently bound to the substrate, so ions could leach from the column [19,20,21,23,24,25].

Phos-tag: this method is considered an IMAC variation. It can be applied to both phosphopeptide and phosphoprotein enrichment. The matrix of this method is composed of a biphasic phosphate binding tag (phos-tag), 1,3-bis(bis(pyridine-2-ylmethyl)amino)propan-2-olato dizinc (II) complex. The matrix carries two zinc ions that are capable of accepting two electrons from a phosphate group (phosphates can donate two oxygen bound electrons) and under neutral pH conditions are strongly bound. In acidic conditions (at pH 3–4), the captured compound can be deprotonated and released. This acidic condition unfortunately also makes the amino acid deprotonated and results in their non-specific binding [20,26].

Metal oxide affinity chromatography (MOAC): for high recovery and specificity, MOAC is the most popular enrichment method. This method depends on the interaction between metal oxide or hydroxide and functional groups such as the phosphate group. Phosphopeptide level enrichment is most commonly based on titanium dioxide (TiO_2_), whereas aluminum hydroxide (Al(OH)_3_) is used for phosphoprotein enrichment. Other metal oxides used in MOAC include, zirconium dioxide (ZrO_2_), gallium oxide (Ga_2_O_3_), ferric oxide (Fe_3_O_4_), niobium oxide (Nb_2_O_3_), stannic oxide (SnO_2_) and hafnium oxide (HfO_2_) [19,21,24].

Sequential elution from IMAC (SIMAC): This method is a combination of MOAC and IMAC methods for phosphopeptide enrichment. The combination of these two methods strengthens binding selectivity and sensitivity. Mono and multi-phosphorylated peptides can be efficiently enriched from this method. However, the technique needs to be coupled with prefractionation to be most efficient in identifying phosphopeptides from complex samples alone [24,27].

Besides the methods described above, other methods in phosphopeptide and phosphoprotein enrichment include polymer-based metal-ion affinity capture (PolyMAC) method, hydrophilic interaction chromatography (HILIC), hydroxyapatite chromatography method, chemical modification method, prefractionations methods and calcium phosphate precipitation method [21,24,28].

Several strategies to improve phosphopeptide quantification have been developed such as stable isotope labeling of amino acids in cell culture (SILAC), which was first developed by Ong and colleagues in 2002 [29]. It is based on the addition of labeled essential amino acids to amino acid deficient cell culture media that results in the incorporation of non-radioactive, stable isotope-containing amino acids into all newly synthesized proteins. Two years later, this group used different stable isotopic forms of arginine in the SILAC method to simultaneously investigate the global dynamics of phosphotyrosine-based signaling events at three different time points of growth factor treatment [30].

Isobaric tag for relative and absolute quantitation (iTRAQ), which uses isobaric reagents to label the primary amines of peptides and proteins was first developed by Ross and colleagues [31]. Another method that was developed by Hsu and colleagues [32] is stable isotope dimethyl labeling, which used formaldehyde to globally label the N-terminus and epsilon-amino group of lysine via reductive amination.

During the last decade, a number of studies have been performed in the development of new strategies to increase the accuracy and the throughput of quantitative phosphoproteome analysis (Table 1).

Song and colleagues developed a pseudo-triplex dimethyl isotope labeling approach coupled with an IMAC enrichment and online reversed phase-strong cation exchange-reversed phase (RP-SCX-RP) multidimensional chromatography/LTQ-Orbitrap mass spectrometry in order to improve the accuracy of large scale quantitative phosphoproteome analyses. This approach is based on labeling two identical samples with light and heavy isotopes and the comparative sample with an intermediate isotope. Employing this strategy to quantify the differences in the phosphoproteomes of hepatocellular carcinoma (HCC) and normal human liver tissues led to a 50% increase in phosphopeptide quantification and a 50% decrease in experimental time (42 h) than the conventional duplex labeling approach giving the same quantification accuracy. They identified 1918 phosphorylation sites from 1033 phosphoproteins and also found novel phosphorylation sites related to progression of HCC such as pT185 for ERK2 and pY204 for ERK1 that were down regulated in HCC [33]. Lin and colleagues combined stable isotope dimethylation labeling coupled with online 3D SCX-TiO_2_/RP using an LTQ-Orbitrap and super-SILAC mix coupled with selected ion monitoring/ accurate inclusion mass screening (SIM/AIMS), in order to develop a strategy for both the discovery and targeted verification of differentially phosphorylated proteins in three HCCs and their nearby tissue specimens. Their workflow led to the identification of 7868 phosphopeptides [34].

Precision medicine has gained a great deal of attention as a next-generation strategy in cancer therapy. Clinical phosphoproteomics as a valuable tool has been widely used in this field; most of these studies have been performed using surgical tissues and patient-derived models. Results from these studies might not be accurate enough and influenced by ischemia occurring during surgery or by alteration in the characteristics of cancer cells during model generation [39]. To improve the clinical applicability of phosphoproteomics, Abe and colleagues used endoscopic biopsy specimens from patients with gastric cancer, as they can be kept fresh (it needs less than a minute to freeze them), therefore having fewer phosphoproteomic changes, to study the phosphosignaling and potentially therapeutic kinases in in vivo conditions. In this study, three tumor biopsies and three normal gastric biopsies were collected at the same time and after immediate snap freezing in liquid nitrogen (in 20 s) were stored at −80 °C. Phosphopeptides were enriched with Fe^3+^ IMAC resin then labeled with tandem mass tag (TMT) reagents followed by LC-MS/MS analysis. Their strategy was able to identify about 10,000 class 1 phosphosites from endoscopic biopsies [10].

### 2.2. Glycoproteomics

Glycosylation is one of the most important posttranslational modifications (PTMs). More than 50% of mammalian proteins are glycosylated [40]. As the most abundant PTM, glycosylation mediates many biological processes including cell adhesion, cell communication and protein stabilization [41,42,43,44,45,46,47,48]. Aberrant glycosylation has been related to several diseases such as immune deficiencies, cardiovascular disease and cancers [49,50,51,52,53,54]. The bio-function of glycosylation has attracted much interest in recent decades. Among glycoprotein analysis techniques, mass spectrometry is considered as the most common approach to date due to its high sensitivity and capability to provide structural information [55,56,57,58]. Two major strategies, known as “bottom-up” and “top-down”, are utilized for a comprehensive glycoproteomic analysis [59]. However, glycoproteomics remains a great challenge due to (i) the high microheterogeneity of glycoproteins, (ii) glycoproteins are in low abundance relative to normal proteins and (iii) the ionization efficiency of glycopeptides is low in the presence of other peptides. Therefore, enrichment, derivatization and separation techniques have been widely employed to enhance glycoprotein/glycopeptide identification and quantitation.

The two most common types of protein glycosylation are O-linked and N-linked glycosylation [17]. O-linked glycosylation is initiated by sequentially attaching glycans to the hydroxyl oxygen of serine/threonine residues, forming various cores where the final form can be either sialylated, fucosylated or both. In N-linked glycosylation, fourteen sugar preassembled blocks are transferred co-translationally to the amide group of an asparagine residue. N-glycans contain a common pentasaccharide core region consisting of three mannose and two N-acetylglucosamine (GlcNAc) subunits. This can be further modified by the addition of terminal Gal (galactose), GlcNAc, fucose and sialic acid moieties [60].

Glycoproteomics methods are divided into two groups including glycoprotein and glycopeptide-based analyses (Figure 1). The glycoprotein-based approach is used to study the primary structure of glycoproteins and is suitable for identification of glycosylation sites. In this approach, glycoproteins are enriched using various separation methods, such as size exclusion chromatography, chemical immobilization by solid phase extraction, ion exchange and lectin affinity chromatography. Enriched glycoproteins are then subjected to protease digestion to obtain peptides which are subsequently analyzed by mass spectrometry (MS) [61]. In the glycopeptide-based approach, proteins are digested either enzymatically, chemically or both, glycopeptides are enriched and then finally deglycosylated and sequenced by mass spectrometry analysis [62].

The biological importance of glycoproteins has driven the development of novel, sensitive separation and detection strategies. We herein summarize the research performed to improve the existent glycoproteomics methods in GI cancer research (Table 1).

Zhou and colleagues employed a novel fluorescence-based multiplexed proteomics (MP) technology, which included two-dimensional gel electrophoresis (2-DE) followed by the fluorescence staining of glycoproteins and their identification using mass spectrometry to investigate the glycoproteomes of three common HCC cell lines including Chang (non-tumor human liver cell line), Hep3B (non-metastatic HCC cell line) and MHCC97H (highly metastatic HCC cell line) in order to establish a glycoprotein database of human liver cells. This strategy led to the identification of 80 glycoproteins from these three cell lines [35].

In order to investigate N-glycosylation sites on secreted proteins of human HCC, Cao and colleagues used two glycopeptide enrichment methods, namely hydrophilic affinity (HA) and hydrazide chemistry (HC), followed by nano-LC-ESI-MS/MS analysis. This strategy showed the capability of capturing more low abundant proteins and identified about 300 glycosylation sites from 194 glycoproteins [36].

In 2014, Sun and colleagues further provided the largest dataset of quantitative information about N-glycoproteomes of human HCC and healthy liver tissues at that time. They developed an integrated workflow, combining digestion with multiple proteases (trypsin, Glu-C and chymotrypsin) with solid phase-based labeling in one reaction vessel to compare the N-glycoproteome of HCC with that in healthy human liver tissues. While using multiple digestion steps was reported to improve protein identification, it was already used in the qualitative analysis of the proteome [63]; Sun and colleagues in this study used this approach for the first time for the quantitative analysis of the glycoproteome. In all, they quantified 2329 N-glycosites on 1052 N-glycoproteins; of those, about 455 N-glycoproteins were found with significant changes between HCC and normal human liver tissues [37].

N-glycosylation occurs with low stoichiometry and is highly dynamic in different glycoproteins. Moreover, a large number of unglycosylated peptides interfere with glycopeptide signals in mass spectrometry (MS) analysis. These two problems are challenging in glycoprotein identification [38], therefore, developing an efficient enrichment strategy is of utmost importance in glycoproteomics research. Several enrichment methods have been widely used, such as hydrazide chemistry which captures glycoproteins by covalent bonding, lectin-based strategies based on the specific recognition between lectin and sugar, TiO_2_ and silica-based materials and Zwitterionic hydrophilic interaction liquid chromatography (ZIC-HILIC) [64,65,66] which is one of the most efficient methods.

Jiang and colleagues optimized the ZIC-HILIC enrichment method and developed a seamless workflow including protein digestion, desalting and N-glycopeptide enrichment in a 96-well plate, in order to reduce the experiment time and sample loss. Combining the 2D fractions (using RP, SCX and HILIC) and multi-parallel enrichment in glycoproteomic analysis of three HCC cell lines with different metastatic potentials (Hep 3B, MHCC97L and MHCCLM3) led to the identification of 5466 N-glycosites in 2383 glycoproteins, which was the largest dataset on these three HCC cell lines [38].

## 3. Phosphoproteomics in GI Cancers

In the past, the activities of a limited number of kinases were assessed using biochemical methods. Nowadays, recent advances in large-scale phosphoprotein identification have paved the way for investigating different aspects of cancer including the mechanisms underlying cancer initiation and progression, biomarker discovery, drug resistance mechanisms and ultimately identification of therapeutic targets. In this review, we summarize recent findings of phosphoproteomic analyses in several types of epithelial based-GI cancers and the selected biomarkers are shown in Table 2.

### 3.1. Colorectal Cancer

Colorectal cancer (CRC) is the fourth leading cause of cancer, which is expected to account for about 2.2 million new cases and 1.1 million deaths by 2030 worldwide [81]. Almost 25% of the patients developed metastatic disease (mCRC) at diagnosis, and about half will develop metastasis at a later stage [82]. The five year survival rate is less than 50% in advanced stages and more than 90% in early stages [83]. Therefore the identification of biomarkers for early diagnosis along with therapeutic targets and mechanisms underlying drug action and resistance is a matter of importance amongst researchers. Intriguingly, the effect of context heterogeneity such as genetic variation was also investigated in some analyses.

As mentioned before, cancer associated aberrant protein phosphorylation is important in cancer related processes. One of the widely investigated kinases is Src family of kinases (SFKs). The nine members of the SFK including Src, Lck, Lyn, Frk, Blk, Fyn, Yrk, Fgr, Hck and Yes are membrane-associated, non-receptor tyrosine kinases that play a key role in various signal transduction pathways. All of these members share a conserved domain structure containing SH3, SH2, tyrosine kinase (SH1) domains and N-terminal SH4 domain. SFKs play an important role in cell growth, motility and survival; dysregulation of this family has been observed in human cancers [84]. Therefore, investigating the oncogenic activity of this family in different types of cancers has gained a great deal of attention. Dubois and colleagues used SILAC phosphoproteomics to reveal the mechanism underlying YES oncogenic signaling in CRC [67], in which its role in unique signaling is related to CRC progression as observed previously [85]. Their results indicated that micro-domain-associated cell adhesive components, receptor tyrosine kinases and upstream regulators of RAS/MAPK signaling (EGFR, SHC and SHP2) are phosphorylated specifically by YES and no other member of the Src family. This role of Yes was found to rely on palmitoylation of its SH4 domain which helps Yes localization in cholesterol-enriched membrane micro-domains [67]. Furthermore, Vasaikar and colleagues carried out comparative proteomic and phosphoproteomic analyses between 106 paired colon cancer tumor and normal nearby tissues to identify potential therapeutic targets. Phosphoproteomic analysis resulted in the identification of 63 phosphosites on 50 proteins as cancer-associated phosphosites and showed that Rb, a known tumor suppressor is phosphorylated in colon cancer and its phosphorylation is associated with increased proliferation and decreased apoptosis, therefore suggesting Rb phosphorylation as a driver and therapeutic target in colon cancer [68].

KRAS mutations are found in one-third of CRC tumors. Patients with these mutations have shown different responsiveness to treatment [86]. Hammond and colleagues used proteomics and SILAC phosphoproteomics to investigate the effects of KRAS common mutations, including G12D vs. G12V or G12D vs. G13D on KRAS signaling in isogenic human SW48 CRC cell-lines [87]. Their results showed that each mutation generates a distinct signaling network signature which affects the expression and phosphorylation of genes with a role in promoting oncogenic CRC signaling and tumorigenesis, the pre-eminent example being double cortin-like kinase-1 (DCLK1), which was overexpressed in all variants of codon 12 KRAS mutant cells but not in G13D cells [87].

Cancer progression is correlated with cell invasive behavior, angiogenesis, resistance to apoptosis and migration. Protein phosphorylation plays an important role in all of these processes. Therefore, finding the changes is highly valuable. Since a large number of pathways are involved in these processes and their signaling crosstalk; high-throughput phosphoproteomics is a strong tool for investigating these processes [88]. Schunter and colleagues performed comparative phosphoproteomics between patient-matched SW480 (Stage II) and SW620 (Stage III) cell lines to investigate the signaling alteration in cancer progression. In this study, they used a combination of IMAC and TiO_2_ enrichment strategies, which resulted in more complete phosphoproteome coverage than each strategy alone. Their results indicated that several biological processes including cellular adhesion, cytoskeletal structure, mitosis, mRNA transport and translation were differentially regulated between primary and metastatic tumor cell lines. Moreover, the greatest increase in phosphorylation of Ser2 of eIF2S2 without any changes in eukaryotic initiation factor 4E (eIF4E) expression was observed in the SW620 cell line. Altogether, they suggested that metastatic cells deregulate mRNA stability and translational efficiency with constitutive changes to the phosphoproteome [89]. In another study, the phosphorylation ratio of serine, threonine and tyrosine amino acids was identified as 90%, 10% and <1% respectively (8). Dysregulated tyrosine phosphorylation has been reported to be progressively increased in colorectal tumors during its progression and metastasis (9). In this regard, Lin and colleagues employed a comparative phosphotyrosine proteomic study of two CRC cell lines with different progression abilities, SW480 and SW620, which were obtained from a primary colorectal adenocarcinoma and lymph node metastasis of a single patient, respectively, in order to find prognosis biomarkers and establish an accurate risk assessment for stage II CRC patients. Their results showed a total of 103 pTyr peptides from 98 proteins with differential levels between these two cell lines and suggested that CDK1 pTyr15 (3.3-fold greater level in SW480 cells) is an indicator of disease-free survival probability for stage II CRC patients [69].

Hitherto, there are no predictive biomarkers for standard chemotherapeutic treatments and about 50% of the CRC patients do not respond to first-line treatment [70,90]. Therefore, finding biomarkers for drug response and also mechanisms of drug action and resistance is highly valuable, in order to improve drug efficacy and precision medicine. One of these important issues is oxaliplatin (oxPt) resistance in CRC. To study the mechanisms underlying oxPt resistance in the human CRC cell line, Rasmussen and colleagues used phosphoproteomics combined with proteome and transcriptome profiling with a focus on miR-625-3p as a predictive marker for oxPt-resistance. Their results showed that miR-625-3p overexpression induces oxaliplatin resistance by targeting MAP2K6 and consequently reducing MAP kinase signal transduction after genotoxic stress conveyed by the MAP2K6–MAPK14 pathway and leading to a reduced p38-mediated apoptosis and increased cell cycle progression signals [70]. In another study, Abe and colleagues combined IMAC-based global phosphoproteomics with deep phosphotyrosine proteomics to compare the phosphoproteome of cetuximab sensitive (LIM1215 and DLD1) and resistant CRC cell lines (HCT116 and HT29) to find active kinase candidates in resistant cell lines as potential therapeutic targets. Results of their analyses showed that 15 kinases were differentially activated between HCT116 and two sensitive cell lines. Intriguingly, no information on genomic mutation was identified in the COSMIC database for 13 out of 15 identified kinases until 2017, which demonstrated the importance of phosphoproteomic studies where genomic studies are of limited value; however, by Nov 2020 the information about the genomic mutation of four additional kinases has been added to the COSMIC database and still nine kinases remain with no matching record for genomic mutation in the COSMIC database. Moreover, their analysis showed that SRC-PRKCD cascade was activated in HCT116 cells and treatment with an SRC inhibitor inhibited cell proliferation [71]. Previous reports showed that activation of Src family kinases induces the nuclear translocation of EGFR and consequently its endurance to various stresses [91]. Moreover, other members of the Src family were also up-regulated in HCT116 cells which led to the identification of Src family kinases as potential therapeutic targets in the case of cetuximab-resistant CRC [71].

In another study, Kubiniok and colleagues studied the cancer cell responses to RAF inhibitors, and compared temporal changes in the phosphoproteome of two colon cancer cell lines that respond differently to vemurafenib (an RAF inhibitor), Colo205 and HCT116. In fact, phospho-ERK1/2 (pERK) was inhibited in Colo205 cells after treatment with the RAF inhibitor, while it is induced in HCT116 cells. They identified 37,910 phosphorylation sites, of those, 660 were dynamically modulated following vemurafenib treatment. Most of these dynamic phosphorylations were directly related to phospho-ERK profile of the two cell lines. Moreover, their results led to the identification of novel ERK downstream substrates such as several Rho GEFs and Rho GAPs, showing that vemurafenib specifically targets RAF with low off-target activity on kinases or phosphatases [92].

More interestingly, Taniguchi and colleagues employed gene expression, protein expression and protein phosphorylation to explore the mechanisms underlying the improved clinical activity using an anti-EGFR antibody (panitumumab) followed by an anti-VEGF antibody (bevacizumab) (PB) rather than using bevacizumab followed by panitumumab (BP) in patients with RAS wild-type (WT) metastatic colorectal cancer (mCRC). Their results showed the reduced phosphorylation status of key cancer-related signaling proteins, such as EGFR and EPHA2 in both PB and BP groups compared with the vehicle control group (which received saline twice-weekly). However, this reduction was greater in PB than in BP. Moreover, the level of RSK which induces pEPHA2, showed that PB had a negligible effect on RSK1 protein and RSK S380-phosphorylation (pRSK) compared to an increased pRSK level in BP. In addition, several differences in gene expression level were observed, such as reduced lipogenic and hypoxia related genes in PB. In all, their results indicated numerous mechanisms at different levels, including gene expression, protein expression and phosphorylation are involved in the greater efficacy of PB than BP [93].

Stewart and colleagues in 2019 used an antibody-based phosphoproteomic platform to explore the changes in 50 phosphoproteins upon treatment with seven targeted anticancer drugs in 30 cell lines from three different cancers derived from the same oncogene (KRASMT), including non-small cell lung cancer (NSCLC), pancreatic adenocarcinoma (PDAC) and CRC cells. Their results indicated significant differences between NSCLC and CRC and for the first time reported that MEK is differentially phosphorylated by PI3K inhibitors in NSCLC and CRC cell lines [94].

### 3.2. Gastric Cancer

Gastric cancer (GC) is the fifth most common cancer and the third leading cause of cancer related death worldwide where one million new cases of GC are diagnosed yearly. Perioperative chemotherapy and surgery improve overall survival; however, the 5-year overall survival is still less than 50% [95]. There is an urgent demand for novel therapeutic agents. Tremendous efforts have been devoted to find effective drugs; however, drug resistance in some patients is still a grave challenge in this area.

GC is a multifactorial disease with several environmental and genetic risk factors such as infection with *Helicobacter pylori (H. pylori)* [96]. Finding related signaling pathways would be highly valuable for improved treatment and precision medicine. Phosphoproteomic analysis has gained a great deal of attention to help these demands and many researches have been done during the last decade.

*H. pylori* is a Gram-negative bacterial pathogen that colonizes the gastric epithelium of approximately half of the world’s population. Infection with *H. pylori* causes chronic inflammation and is the major risk factor for developing GC [97]; therefore, finding the mechanisms underlying *H. pylori*-induced GC is highly important. It has been shown that *H. pylori* regulates several signaling pathways in gastric epithelial cells, including epidermal growth factor receptor (EGFR) activation, which is overexpressed in GC and involved in tumor cell motility, invasion and metastasis [98]. Moreover, *H. pylori* infection is reported to increase spermine oxidase (SMOX) expression and its enzymatic activity induces oxidative stress and DNA damage, and consequently apoptosis. However, sustained *H. pylori* infection generates a subpopulation of gastric epithelial cells which are resistant to apoptosis [99]. *H. pylori* infection induces EGFR phosphorylation and inhibits apoptosis in epithelial cells [98]; however, the mechanism is not clear. Chaturvedi and colleagues in 2014 compared the phosphoproteome of gastric epithelial cells obtained from *H. pylori*-infected mice with attenuated EGFR activity, wild-type mice or infected cells incubated with EGFR inhibitors or deficient in EGFR. Results revealed increased EGFR and ERBB2 signaling and indicated that pEGFR is required to increase SMOX expression and DNA damage in *H pylori*-infected gastric epithelial cells and reduces apoptosis through activating ERBB2 and AKT. Moreover, pEGFR staining of two independent tissue microarrays containing each stage of disease, from gastritis to carcinoma showed that pEGFR is increased in gastritis and atrophic gastritis, but not in intestinal metaplasia (IM), dysplasia or GC. This indicates that EGFR signaling and associated SMOX induction could be involved in initiation of gastric carcinogenesis [100].

Adenocarcinoma is classified into two groups according to differences in morphology, epidemiology, pathology and genetic profiles. The first group is well-differentiated or intestinal type gastric cancer (IGC) which accounts for 60% of cases and is more common in older patients and is mainly caused by *H. pylori* infection. The second group is diffuse-type gastric cancer (DGC) which accounts for 30% of cases where its pathogenicity remains undefined and no effective treatment is yet available [101]. In 2019, Tong and colleagues performed quantitative phosphoproteomics of 83 diffuse-type gastric cancer (DGC) tumors and their adjacent tissues, which identified 6734 differentially phosphorylated sites between tumors and normal tissues. Moreover, they performed consensus clustering to classify DGC tumors, which resulted in three subtypes with 302 differentially phosphorylated sites which are associated with different overall survival (OS) and sensitivity to chemotherapy. Furthermore, they found a set of 16 kinases mostly involved in MTOR signaling network which were activated with high frequency and are associated with poor OS [102].

Lee and colleagues compared the phosphoproteome of human GC cells resistant to lapatinib (a dual inhibitor of EGFR and HER2 tyrosine kinases) with parental cells (SNU216) in order to find changes related to lapatinib resistance. Their results revealed 95 differentially phosphorylated proteins (DPPs) that are involved in MET-axis induced activation of phosphatidylinositide 3-kinase (PI3K)/α-serine/threonine-protein kinase (AKT) and mitogen-activated protein kinase (MAPK)/extracellular signal-regulated kinase (ERK) signaling pathways in SNU216-LR. Moreover, the combination of lapatinib with NVP-BEZ235 (PI3K/mTOR inhibitor), selumetinib (MEK inhibitor), saracatinib (SRC inhibitor) and crizotinib (MET inhibitor) restored lapatinib sensitivity [103].

### 3.3. Pancreatic Cancer

Pancreatic cancer (PC) is the eleventh most common type of cancer and seventh leading cause of cancer-related deaths globally with about 458,918 new cases and 432,242 deaths in 2018. PC is one of the cancers with the poorest prognosis, with the five year survival rate close to 9% [104]. Early systemic spread and drug resistance are the main reasons for these dismal outcomes [105]. During the last decade, phosphoproteomics has been employed by researchers as a reliable tool to explore molecular mechanisms, biomarker discovery and investigating the drug resistance mechanisms in PC.

Approximately 50% of PC cases are diagnosed with metastases to distal sites, of which liver, lung and peritoneum are the most common sites [106]. Therefore, finding the mechanisms underlying metastases could aid in the discovery of potential therapeutic targets and reduce mortality rates. In this regard, Kim and colleagues performed comparative proteomic and tyrosine phosphoproteomic analyses between cells isolated from three metastases sites of PC including liver, lung and peritoneum from a single patient to explore differentially activated cellular signaling pathways as a consequence of genetic variations, as it had been reported by previous studies that organ-specific metastases are derived from different subclones of primary tumor with genetic heterogeneity. Their results showed a unique pattern of protein expression and tyrosine kinases activities in each metastatic lesion. Moreover, treatment with R428, a tyrosine kinase inhibitor targeting Axl receptor tyrosine kinase indicated different effectiveness in inhibiting cancer cell proliferation both in cell lines and mouse model, which was due to differences in hyperphosphorylation of Axl on Tyr634, Tyr702 and Tyr703 across the three metastatic cell types. These results indicated that a combination of agents should be used for targeting the features of different subclones [107].

Solid tumors are a heterogeneous mixture of cancer cells and stromal cells where crosstalk between them regulates many hallmarks of cancer. Demircioglu and colleagues used transcriptomic, proteomic and phosphoproteomic approaches to explore the mechanisms by which cancer associated fibroblasts (CAFs) regulate malignant cell metabolism. Their results showed that CAFs regulate the metabolism of tumor cells by focal adhesion kinase (FAK) through regulating the expression of chemokines; Ccl6 and Ccl12, which in turn regulate malignant cell Ccr1/Ccr2 activity and activation of protein kinase A (PKA). Low stromal FAK expression leads to chemokine production and protein kinase A activation and consequently enhances glycolysis in tumor cells. Moreover, a reduced level of FAK was found to be associated with a reduced survival rate [72]. Moreover, Tape and colleagues used heterocellular multivariate phosphoproteomics to investigate the oncogenic KRAS (KRAS^G12D^) cell-autonomous, nearby non-tumor cells, and reciprocal signaling in pancreatic ductal adenocarcinoma (PDAC; an extremely heterocellular malignancy composed of mutated tumor cells, stromal fibroblasts, endothelial cells and immune cells). Heterocellular investigation revealed SHH, AKT and IGF1R/AXL as therapeutic targets to perturb KRAS^G12D^ signaling along with identified targets in PDAC cells screening alone; MEK, MAPK and CDK. Their results implicated the importance of reciprocal signaling in the regulation of apoptosis, proliferation and mitochondrial performance in tumor cells. Their findings also demonstrated the importance of understanding cancer as a heterocellular disease and considering the heterocellular process to study oncogenic signaling. In this study, they presented a heterocellular multivariate phosphoproteomic workflow which could be used to investigate in vitro oncogenic reciprocal signaling [108].

There is ample evidence that the high mutation rate of oncogenic KRAS may be responsible for driving PDAC initiation and progression. Chen and colleagues employed unbiased global gene expression and phosphoproteomic analyses to study the pivotal role of oncogenic KRAS in PDAC development and potential compensatory pathways after acute and sustained KRAS inhibition. While genetic or transcriptional changes were not significant, phosphoproteomic analysis indicated significant changes in cell signaling networks. Results of phosphoproteomic profiling of KRAS-inhibited cells revealed the mechanisms of resistance to KRAS blockade, including increased focal adhesion plaque structures, adherence properties, and dependency on adhesion for viability in vitro. Their results showed that most PDAC cells tolerated KRAS inhibition through adapting non-genetic and non-transcriptional mechanisms [109].

Cancer stem cells (CSCs) are a less differentiated subpopulation within the heterogenous bulk of tumors which can maintain their self-renewal property and account for therapeutic resistance. Bian and colleagues performed a high-throughput drug screening to identify the effects of selected drugs against tumor-promoting CSCs of PC in 2D and 3D cell cultures. Their results indicated the effectiveness of 1H-benzo(d)imidazol-2-amine-based inhibitor of interleukin-2-inducible T-cell kinase (ITK) (NCGC00188382, inhibitor #1) to inhibit growth and induce apoptosis in vitro and suppress cancer progression and metastasis in vivo. In this study, they used in vitro and in situ phosphoproteomic analysis to explore the polypharmacology of this drug. Results showed that this agent inhibited TAOK3, CDK7 and aurora B kinases and that TAOK3 signaling is required for tumor initiation and metastasis formation [105].

### 3.4. Hepatocellular Carcinoma (HCC)

HCC is the fourth most common cause of cancer-related death globally [110], which accounts for about 600,000 deaths each year [111]. Most of the HCC cases are diagnosed at the advanced stage and despite the advances in clinical treatments, such as resection, the mortality rate remains high due to recurrence and metastases [110]. Furthermore, current drugs such as sorafenib showed limited efficacy [112]. Therefore, there is an urgent need to find the progression mechanisms and therapeutic targets.

Tian and colleagues employed an off-line high-pH HPLC separation coupled with multi-step IMAC and LC–MS/MS to investigate the phosphoproteome of a metastatic hepatocellular carcinoma cell line (MHCC97-H) to find the main phosphoproteins in HCC metastasis. Their results led to the identification of 2930 phosphoproteins which were involved in several biological processes. Comparison of these findings with a published phosphoproteome of a normal liver sample showed that phosphoproteins involved in the spliceosome pathway, such as U2 small nuclear RNA auxiliary factor 2 (U2AF2), eukaryotic initiation factor 4A-III (EIF4A3), cell division cycle 5-like (CDC5L) and survival motor neuron domain containing 1 (SMNDC1) were only identified in the MHCC97-H cell line. Their results highlighted the role of phosphorylation of spliceosome related proteins in HCC metastasis [105]. More interestingly, Ren and colleagues for the first time performed in vivo quantitative phosphoproteomics combined with computational strategies to investigate phosphoproteome changes in an HBx-transgenic mouse model HCC and to identify predominant kinases as potential targets for cancer therapy and to explore related molecular mechanisms. Their phosphoproteome analysis yielded 22,539 phosphorylation sites from 5431 proteins. Of those, Src family kinases (SFKs), PKCs and Rho associated protein kinase 2 (ROCK2) identified as the predominant kinase families in HCC and implicated the role of Src Ser17 in cell migration of HCC and ROCK2 kinase activity [113].

Viral hepatitis B (HBV) and C (HCV) infections are the main risk factors for HCC and about 80% of the HCC patients are infected by these viruses [114]. Therefore, finding the mechanisms linking these infections with HCC is of great importance among researchers. In this regard Lu and colleagues in 2016 performed a comparative phosphoproteomic analysis between HCC cell lines with and without HCV to explore the role of HCV in core HCC pathways. Their results showed that differentially phosphorylated proteins were involved mainly in the metabolic insulin response, cytoskeletal dynamics impacting cell growth, viral-mediated host mRNA transcription and phosphatidylinositol-3-kinase/protein kinase B (PI3K/AKT)-driven oncogenic survival [115]. Furthermore, Abdallah and colleagues in 2017 performed a quantitative phosphoproteomic analysis to study the signaling pathways activated by the HCV core in hepatoma cells. Their results indicated that 31 proteins were differentially phosphorylated in HCV core expressing cells. These proteins were involved in metabolism, insulin receptor signaling, protein transport, cell death, cellular component organization and translation. Among hyperphosphorylated proteins, those involved in translation, in particular 4E-BP1, showed to be a tumor-specific target of the HCV core [116].

Jiang and colleagues carried out proteomic and phosphoproteomic profiling of 110 tumor tissues from early stage cases of HCC related to HBV infection and non-tumor paired tissues. Based on proteomics data analysis, they divided the cohort into the three groups including S-I, S-II and S-III, of which S-III was considered to be associated with the lowest overall rate of survival and the greatest risk of a poor prognosis after first-line surgery. Sterol O-acyltransferase 1 (SOAT1) was introduced as the specific signature of S-III which its inhibition by avasimibe significantly reduced tumor size [117].

Drug resistance remains a main issue of therapy failure which drastically has increased cancer related-mortality. One of the main chemotherapeutic agents which have been widely used for HCC treatment is 5-fluorouracil (5-Fu). However, most HCC patients respond poorly to 5-FU due to the development of chemoresistance. In a study, Liu and colleagues compared the proteome and phosphoproteome of 5-FU resistant Bel/5-Fu cell line and its parental Bel cell line using stable isotope dimethyl labeling combined with high-resolution mass spectrometry to study the molecular mechanism underlying causing 5-Fu resistance. Their results led to the identification of 8272 unique proteins and 22,095 phosphorylation sites. They showed that phosphorylation levels of PLCβ3 pS1105, which is involved in the GnRH signaling pathway, and protein levels of SRC and PKCδ, which could phosphorylate PLCβ3 at Ser1105, were increased in Bel/5-Fu cells compared to Bel cells. Knockdown of these proteins led to the susceptibility of Bel/5-Fu cells to 5-Fu [118]. Moreover, Chen and colleagues, in order to find the mechanisms underlying sorafenib resistance in advanced-stage hepatocellular carcinoma, performed comparative quantitative phosphoproteomics between parental and sorafenib-resistant HuH-7 cells. A total of 1500 differential phosphoproteins were identified, of those, 533 were significantly up-regulated in resistant cells. Bioinformatics analysis showed the involvement of several pathways including cell adhesion and motility, cell survival and cell growth. Their results showed that sorafenib-mediated protein tyrosine kinase inhibition could lead to the activation of alternative cellular pathways such as the Akt, mTOR and FAK signaling pathways and showed that suppressing EphA2 increases its susceptibility to sorafenib. These observations suggested that targeting of EphA2 along with sorafenib treatment helps to overcome sorafenib resistance and resulted in better management of advanced hepatocellular carcinoma [73].

More interestingly, phosphoproteomics has also been used to investigate the efficacy of conventional, as well as novel developed drugs. In this regard, Melas and colleagues in 2013 investigated phosphoproteome changes in 3 HCC cell lines upon treatment with eight drugs including lapatinib, gefitinib, erlotinib (EGFR inhibitors), sorafenib (inhibitor of VEGFR, PDGFR and of Raf kinases C-Raf and B-Raf), vandetanib (VEGFR and EGFR antagonist), sunitinib (PDGFR and VEGFR kinase inhibitor), dasatinib (multi-BCR/ABL and Src family kinase inhibitor), and bortezomib (proteasome inhibitor) for unresectable HCC, in order to identify the phosphoproteomic signatures that are predictive of drug efficacy. Their results showed that measuring the activation level of 16 key phosphoproteins under six stimuli could be used to predict drug efficacy, among them inhibition of AKT under TGFα and AKT under HER are indicative of a clinically failed drug, while ERK12 under HGF is an effective drug (accuracy 80%) [119]. Furthermore, Fernández-Varo and colleagues performed MS-based phosphoproteomics of hepatic tissues from 110 Wistar rat model of HCC following treatment with cerium oxide nanoparticles (CeO_2_NPs), a novel antioxidant agent, to study its potential therapeutic properties. Their results showed that CeO_2_NPs effectively reduced tumor progression and significantly increased survival in HCC rats. This mainly affects phosphorylation of proteins involved in cell adhesion and RNA splicing [120].

## 4. Glycoproteomics in GI Cancers

Exploring glycoproteins on the surface of the cellular membrane, secretome and body fluids are of interest among researchers. Currently, several glycoproteins are used as cancer biomarkers including CA 19-9 for PC, CA 125 for ovarian cancer and CA 15-3 for breast cancer [121]. However, they often have poor specificity because the detected glycoprotein is also found in elevated levels in nonmalignant conditions. Reliable, accurate biomarkers for early detection provide the highest likelihood of curative therapy and survival.

Fortunately, during the last decade, numerous glycoproteomic studies have been carried out in order to find reliable biomarkers (Table 2). Although a wide range of glycan epitopes are found in human GI, which might be altered during the process of carcinogenesis and cancer progression, most of the studies investigated the changes in O-GlcNAcylation, truncated simple O-glycans, N-glycan branching, sialylation and fucosylation. In the following section, we describe recent glycoproteomic studies on several of GI cancers, including: CRC, GC, HCC and PC.

### 4.1. O-Linked Glycosylation

Peiris and colleagues used lectin *Helix pomatia* agglutinin (HPA) coupled to 2-dimensional gel electrophoresis with mass spectrometry to explore O-linked glycosylation changes in tissue samples of colorectal cancer lymph node metastasis. Moreover, the correlation of O-linked glycosylation with P53 in KRAS mutation and the Carcinoembryonic Antigen (CEA) status as the biomarkers of CRC were evaluated. Their results showed early changes in O-linked glycosylation associated with metastatic colorectal cancer which were not affected by p53 and KRAS mutations. In addition, annexin 4 was observed to be the major carrier of O-linked glycans in lymph nodes from CRC samples and have shown that the level of annexin 4 is positively associated with CEA status, a predictor of aggressive metastatic phenotype and poor survival rate [74].

**Figure 2:**
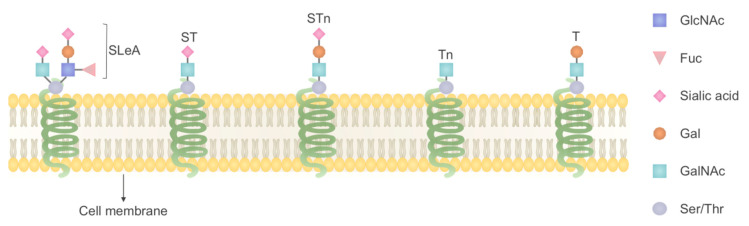
Glycan alteration in GI cancer, one common feature of GI cancers is the overexpression of Tn antigen (GalNAcα1-Ser/Thr), T antigen (Galβ1-3GalNAcα1-Ser/Thr), STn (Neu5Acα2-6GalNAcα1-Ser/Thr), ST (Neu5Acα2-3Galβ1- 3GalNAcα1-Ser/Thr) and also sialyl-Lewis A (SLeA).

O-N-acetylgalactosamine (O-GalNAc) is a common type of O-glycosylation, and its addition to proteins (hydroxyl groups of selected serine and threonine residues) is catalyzed by a family of enzymes named GalNAc-transferases (GalNAc-Ts). O-GalNAc glycosylation of proteins has been reported to be changed in cancers [60], considering this fact, Lavrsen and colleagues using transcriptomics analysis showed that polypeptide N-acetylgalactosaminyl transferase 6 (GalNAc-T6) expression is essential for the acquisition of oncogenic phenotype in the LS174T colon cancer cell line. A differential O-glycoproteomic analysis was then applied to identify GalNAc-T6 targets in colon cancer cell lines with a knockout/rescue system for GALNT6. Their results led to the identification of a small set of GalNAc-T6–specific targets such as MIA3 (melanoma inhibitory activity family, member 3), ephrin B6 receptor (EphB6) that were involved in several biological processes such as cell–cell recognition, cell–matrix adhesion and migration. It has been suggested that the overexpression of GalNAc-T6 is an early event during cancer development [75]. Expression of truncated O-glycans and the structures T (Galβ1–3GalNAcɑ1-O-Ser/Thr), ST (Neu5Acα2-3Galβ1- 3GalNAcα1-Ser/Thr), STn(NeuAcɑ2–6GalNAcɑ1-O-Ser/Thr) and Tn (GalNAcɑ1-OSer/Thr) has been reported in cancer cells (Figure 2), but not in healthy cells [4]. Therefore, these are considered as characteristic phenotypes of cancer cells. In a study by Campos and colleagues, two O-glycoproteins, CD44 and GalNAc-T5 with O-glycan STn were observed in both GC cells and sera of patients with GC, and indicated as potential biomarkers in gastric cancer [76].

More recently, Fernandes and colleagues explored the sialyl-Lewis A (SLeA)-glycoproteome of six GC cell models with emphasis on glycoproteins showing affinity for E-selectin to identify potential biomarkers. Sialylated-Lewis (SLe) antigen is a terminal glycoepitope of glycoproteins present at the cell surface, sialyl-Lewis A isoform (SLeA) is reported to be overexpressed in gastric tumors and mediates the adhesion of tumor cells to endothelium of blood vessels, entering into bloodstream and homing into the distant organs. Their results showed that nucleolin (NCL)-SLeA glycoforms are expressed in primary tumors and metastases and is suggested to be a biomarker of the worst prognosis in GC [77].

### 4.2. N-Linked Glycosylation

N-linked glycoproteins which include transmembrane proteins, cell surface proteins, and secreted proteins are a highly interesting group of proteins for clinical and cancer biological studies, since the expression and glycosylation pattern of these group of proteins is changed in several cancer types [122]. Pan and colleagues carried out a quantitative glycoproteomics analysis to compare protein N-glycosylation between pancreatic tumor tissues, normal pancreas and chronic pancreatitis tissues. They identified several glycoproteins with elevated N-glycosylation levels correlated with pancreatic cancer, including mucin-5AC (MUC5AC), carcinoembryonic antigen-related cell adhesion molecule 5 (CEACAM5), insulin-like growth factor binding protein (IGFBP3) and galectin-3-binding protein (LGALS3BP), which were involved in several well-known pancreatic cancer pathways such as: TGF-β, TNF, NF-kappa-B and TFEB related lysosomal changes [123]. The majority of serum/plasma N-glycoproteins are produced in the hepatobiliary system and an unhealthy situation in the liver, such as HCC results in aberrant serum/plasma N-glycome [11], therefore exploring these alterations in HCC patients is highly valuable in order to identify potential biomarkers. Chang and colleagues investigated the dynamics of N-glycoproteome in plasma samples from patients with HCC, cholangiocarcinoma (CCA), or combined HCC and CCA (cHCC-CCA) to find potential biomarkers related to disease diagnosis and progression; totally 57 differentially expressed proteins were identified, of those, C3 and APOC3 proteins were associated with the tumor stage, tumor grade, recurrence-free survival and overall survival of HCC. In particular, the level of complement C3 with Man5, Man6 or Man7 glycoforms at Asn85 was found to be correlated with HCC tumor grade, even more than α-fetoprotein (a well-known HCC biomarker). Moreover, C3 bearing Man5 or hybrid glycoforms were associated with post-surgery prognosis of HCC even stronger than the whole level of complement C3. In addition, complement C3 with hybrid glycoforms were related with the mortality rate of HCC [11]. In another study, Zhang and colleagues, in order to identify potential serum N-linked glycoproteins related to HCC recurrence, used lectin affinity chromatography combined with enzyme-catalyzed ^18^O_3_− or ^16^O_3_− labeling and screened serum samples from 100 early-stage HCC patients; results were validated using immunohistochemical staining in 193 HCC tissues. They found serum core fucosylated quiescin sulfhydryl oxidase 1 (cf-QSOX1) as a reliable biomarker for post-operative recurrence of HCC. QSOX1 in HCC tissues was positively correlated with good patient outcomes. Further functional analysis showed that overexpression of QSOX1 increased apoptosis and decreased the invasive capacity of HCC cells and lung metastasis in nude mice models with HCC. Furthermore, core-fucosylated glycan at Asn-130 was shown to be essential for inhibitory effect of QSOX1 on the invasive and metastatic potential of HCC [78].

### 4.3. Whole Glycoproteome

In order to find serum biomarkers for early detection of HCC using whole glycoproteomics analysis, Block and colleagues developed a targeted glycoproteomic methodology and investigated the released oligosaccharides from serum glycoproteins. They found several glycoproteins with altered glycosylation in animal models (woodchucks) of HCC, of those; Golgi protein 73 (GP73) was both elevated and hyperfucosylated, introduced as a potential biomarker of liver cancer. Moreover, observing hyperfucosylated AFP, α-1-acid glycoprotein, α-1-antitrypsin and transferrin in their results, where hyperfucosylation in liver cancer was well established previously, confirmed the efficacy of their approach [79]. In another study, Ang and colleagues compared the serum concentration of haptoglobin (Hp) and its glycoforms in patients with HCC and patients with chronic liver diseases (CLD) without cancer using glycosylation-specific lectins and 2D-PAGE. They found HCC-specific Hp glycoforms namely: Alpha-2, 6-sialylated Hp (S-Hp) and Alpha-1, 6-fucosylated Hp (F-Hp) were associated with tumor progression and suggested serum Hp as a potential biomarker for HCC diagnosis [80]. Moreover, Gao and colleagues employed lectin affinity chromatography (LAC) followed by stable isotope dimethyl labeling, 2D liquid chromatography (LC) separation and mass spectrometric analysis to compare the serum glycoproteins of 40 HCC patients with 40 normal control serum samples and established a quantitative glycoproteomic method to find biomarkers for early HCC diagnosis. About 36 up-regulated and 19 down-regulated proteins in HCC samples were found, among them, up-regulation of FOSL2 in HCC was reported for the first time [124]. In another study, Barefoot and colleagues employed glycoproteomic analysis along with transcriptomic and metabolomic analyses of tissue and serum samples of 40 HCC and 25 liver cirrhosis (CIRR) patients to find mechanistic differences between HCC and CIRR, and potential biomarkers distinguishing HCC from underlying cirrhosis. The activated FXR/RXR pathway was found to overlap across omics in both serum and tissue samples, and have implicated this pathway as the target for biomarker discovery. Finally, their analyses led to the identification of C4A/C4B, KNG1 and HPX glycoproteins as the potential biomarkers which were up-regulated in serum of HCC patients [125].

## 5. Conclusions

Phosphoproteomics and glycoproteomics have proved to be strongly efficient in GI cancer research, yielding important biological surprises even when other omics strategies were not informative. They provide valuable information on the mechanisms of GI cancer initiation, progression, metastasis and drug resistance, moreover, enabling diagnosis biomarkers and therapeutic targets discovery. A large number of differentially expressed proteins were identified in these high-throughput studies; however, only a few proteins were selected for functional analyses, as listed in the Table 2. Further research considering other identified proteins for functional analyses may provide more potential biomarkers, moreover, the selected biomarkers in this study also need further validation in larger populations prior to being applied in a clinical setting. Phosphoproteomics and glycoproteomics in GI cancers are now just emerging from their first phase, where they predominantly focused on patient-derived tumor xenografts (PDTXs), body fluids and cell line models, which are time consuming and expensive, or do not represent in vivo (tumor) characteristics, such as tumor heterogeneity. To fulfill their full potential, these approaches need to employ organoids, which resemble the in vivo environment, and also are easy to manipulate and investigate utilizing high throughput screening, which is difficult to perform in tumor xenografts or slice cultures. On the other hand, considering intratumoral molecular heterogeneity, some rare subpopulation may play a critical role in cancer invasion, metastasis and the evolution of resistance to therapy; therefore, using phosphoproteomics and glycoproteomics at single cell resolution enables and promotes knowing the enemy. While these approaches alone are not enough for the victory, they are essential parts of any overall plan of attack.

## Figures and Tables

**Table 1:** Summary of developed methods for phosphoproteome and glycoproteome analyses in GI cancer.

Study	Developed Strategy	Sample	Results	References
**Phosphoproteomic studies**				
Song et al. 2011	Pseudo triplex stable isotope dimethyl labeling approach coupled with on line RP-SCX-RP LC-MS/MS	Hepatocellular carcinoma (HCC) and normal human liver tissues	1934 phosphopeptides from 1033 phosphoproteins	[33]
Lin et al. 2017	Stable isotope dimethylation labeling coupled with online 3D SCX-TiO2/RP LC-MS/MS and super-SILAC mix coupled with SIM/AIMS	Human HCC tissue	7868 phosphopeptides	[34]
Abe et al. 2020	Fe^3+^ IMAC-TMT labeling-SCX LC-MS/MS	Gastric cancer patients endoscopic biopsy specimens	4034 phosphoproteins	[10]
**Glycoproteomic studies**				
Zhou et al. 2007	Two-dimensional gel electrophoresis (2-DE) followed by the fluorescence staining of glycoprotein-MALDI-TOF-MS/MS	Three Human HCC cell lines	80 glycoproteins	[35]
Cao et al. 2009	Glycopeptide enrichment methods; hydrophilic affinity (HA) and hydrazide chemistry (HC), were used complementarily-LC-MS/MS	Human HCC cells	300 glycosylation sites within 194 glycoproteins	[36]
Sun et al. 2014	HC- multiple protease digestion-dimethyl labeling -SCX-RP LC-MS/MS	Human HCC and healthy liver tissues	2329 N-glycosites on 1052 N-glycoproteins	[37]
Jiang et al. 2019	A multi-parallel enrichment strategy based on the optimized ZIC-HILIC enrichment method assisted by a filter-coated 96-well plate-MALDI-TOF MS	Three HCC cell lines	5466 N-glycosites in 2383 glycoproteins	[38]

**Table 2:** Summary of selected biomarkers for GI cancers.

Protein Name	Sample Type	Method	Clinical Significance	References
**Phosphoproteomics studies**				
Phosphorylated YES (Src family)	CRC cell line	SILAC-HPLC-ESI-MS/MS	Therapeutic target	[67]
Rb phosphorylation	Colon cancer tumor tissues	IMAC–LC/MS/MS	Therapeutic target	[68]
CDK1 pTyr15	CRC cell line	TiO_2_-LC-MS/MS	Prognostic biomarker	[69]
miR-625-3p	CRC cell line	SILAC-TiO_2_-LC-MS/MS	predictive biomarker for oxPt-resistance	[70]
Src family	CRC cell line	IMAC-TMT-LC-MS/MS	Therapeutic targets in cetuximab-resistant CRC	[71]
FAK	Mouse model of PC	TiO_2_-LC-MS/MS	Survival rate	[72]
EphA2	PDAC cell line	SILAC-TiO_2_-LC-MS/MS	Therapeutic target in sorafenib resistant CRC	[73]
**Glycoproteomics studies**				
Annexin 4	lymph node positive CRC tissue samples	Lectin HPA- 2DE - MALDI-MS	Survival rate	[74]
GalNAc-T6	colon cancer cell line	VVA LWAC-nLC- nES-MS/MS	Cancer development	[75]
CD44 and GalNAc-T5, with O-glycan STn	cell line and sear of patient with gastric cancer	VVA LWAC- nLC- nES -MS/MS	Diagnostic biomarker	[76]
Nucleolin (NCL)-SLeA glycoforms	Gastric cancer cell models	anti-SLeA -nLC-nES-MS/MS	Worst prognosis	[77]
C3 with Man5, Man6, or Man7 glycoform at Asn85	HCC patient plasma samples	LC-MS/MS	Indicator of HCC tumor grade	[11]
cf-QSOX1	HCC patient serum samples	LAC- nLC-ESI-MS/MS	Biomarker for postoperative recurrence of HCC	[78]
GP73	animal models (woodchucks) of HCC	LAC-2DE-HPLC-MS/MS	Diagnostic biomarker	[79]
Hp	HCC patient serum samples	LAC-2DE- MALDI- MS/MS	Diagnostic biomarker	[80]

Rb: retinoblastoma, CDK1: cyclin-dependent kinase I, FAK: focal adhesion kinase, CRC: colorectal cancer, EphA2: ephrin type-A receptor 2, cf-QSOX1: core fucosylated quiescin sulfhydryl oxidase 1, GP73: Golgi protein 73, Hp: haptoglobin, HPA: *Helix pomatia* agglutinin, 2DE: two dimensional electrophoresis, nLC: nanoflow liquid chromatography, VVA LWAC: *Vicia villosa* agglutinin based lectin weak affinity chromatography, nES: nano-electrospray, LAC: lectin affinity chromatography.
